# Book Review

**DOI:** 10.1120/jacmp.v7i2.2252

**Published:** 2006-05-25

**Authors:** Jatinder Palta

**Affiliations:** ^1^ Professor and Chief of Physics Department of Radiation Oncology University of Florida Gainesville Florida 32610 U.S.A.

## Abstract

*Brachytherapy Physics*, 2nd ed., edited by Bruce R. Thomadsen, Mark J. Rivard, and Wayne M. Butler, AAPM Medical Physics Monograph #31, Medical Physics Publishing, 2005, ISBN 10: 1‐930524‐24‐2, list price $140 (AAPM member), $175 (all others)

This book contains the proceedings of the Summer School on Brachytherapy, jointly organized by the American Association of Physicists in Medicine (AAPM) and the American Brachytherapy Society (ABS). The last AAPM Summer School on Brachytherapy was held in 1994,^(1)^ so the physical and clinical aspects of brachytherapy have changed significantly since that time. A greater understanding of how to accurately determine source dosimetry parameters has led to many advances either by using theoretical calculations such as Monte Carlo methods or in the design of experimental measurements of brachytherapy dosimetry parameters in terms of phantom composition and dimensions and dosimeter selection and calibration procedures. Advancements have also been made in the analyses of experimental uncertainties. The emergence of transrectal ultrasound—guided permanent prostate interstitial brachytherapy for early‐stage prostate cancer has led to the development and manufacturing of several new low‐energy brachytherapy sources. In response, much effort has been spent gaining a detailed understanding of the dosimetric characteristics of these sources, which is reflected in the volume of publications by the Low Energy Interstitial Brachytherapy Dosimetry subcommittee of the AAPM Therapy Physics Committee, which updated the AAPM TG43 report.^(^ ^2^ ^,^ ^3^ ^)^

A new national source strength standard, the National Institute of Standards and Technology Wide‐Angle Free‐Air Chamber system,^(4)^ has been accepted for the general source strength of such sources. Advances in volumetric imaging, in the capability of treatment‐planning systems to use such images, and in optimization methods have led to greater use of image‐guided brachytherapy techniques. The high‐dose rate (HDR) remote afterloading technique has begun to replace the conventional low‐dose rate (LDR) technique in the delivery of brachytherapy treatments to a variety of clinical sites, such as with gynecologic, prostate, and other intraluminal and interstitial treatments. Accelerated partial breast irradiation techniques using interstitial implants or intracavitary placement of a balloon (MammoSite) are gaining general acceptance in the management of early‐stage breast cancer following lumpectomy. Intravascular brachytherapy emerged as the most popular treatment of coronary vessel stenosis, only to be largely replaced by the use of drug‐emitting stents. Finally, unconventional delivery techniques such as Y90‐impregnated microspheres for treatment of liver metastasis and radiolabeled antibody therapy techniques such as Zevalin and Bexxar for treatment of lymphomas are now delivered at radiation oncology departments instead of at nuclear medicine departments, thus adding to the expectations for an additional knowledge base for clinical brachytherapy physicists.

The proceedings of the 2005 AAPM/ABS summer school could not have come at a more opportune time. Compared with the 1994 volume, the current book provides updated information on source strength calibration methods, brachytherapy source characteristics, and dose calculation formalisms contributed by the authors of relevant AAPM task group reports on these topics. Several well‐written chapters are devoted to the concepts of classical implant systems (Manchester, Quimby, and Paris). Additional chapters describe general clinical applications such as implant design, treatment plan review, and postimplant quality evaluation, which were previously available only in diffused literature. These proceedings have placed a much greater emphasis, and have provided detailed discussions, on clinical topics such as image‐guided gynecologic implants, LDR permanent as well as HDR temporary prostate implants, and accelerated partial breast irradiation using both the interstitial and intracavitary techniques. There are several chapters on advanced 3D image‐based brachytherapy; however, most of the information is limited to the current state‐of‐the‐art in HDR treatment techniques. Prostate brachytherapy, which was covered in a single chapter in the previous summer school proceedings, is now covered in six chapters with a plethora of detailed and clinically useful information. The chapters on regulatory aspects of brachytherapy and brachytherapy facility design provide a broader insight into the topic and contain the most current information on revisions made to the federal regulations on the medical use of brachytherapy sources over the past several years.

The book also attempts to achieve a balance between theoretical studies and clinical applications of brachytherapy. While the authors of the chapters are well‐known researchers in brachytherapy physics, many of the other chapters’ contributors are clinical brachytherapy physicists. This has resulted in a balanced presentation of both the future trends in brachytherapy research and the current status of clinical brachytherapy physics.

Given the diversity in clinical implementation of brachytherapy techniques, it is not surprising that the book is unable to provide discussions on several emerging aspects of clinical brachytherapy applications, including the use of functional imaging techniques such as PET and MRS for brachytherapy treatment planning and delivery, and image‐based tandem and ovoid treatments of cervical cancer, especially in how their applications may change the prescription and treatment planning of such implants. It is also missing discussions on the combined use of brachytherapy and external beam treatments. Finally, the summer school proceedings took a narrower definition of brachytherapy by only including techniques that use sealed sources (with the exception of the microspheres). It does not describe the emerging clinical techniques of radiolabeled antibody therapy. Because demand for such treatment options is directed toward radiation oncology departments, guidelines on source preparation, dosimetry, and delivery techniques are of the utmost importance to clinical physicists. These omissions notwithstanding, the second edition of *Brachytherapy Physics* provides an update and detailed information on brachytherapy dosimetry principles and clinical techniques that could not have come at a more opportune time. These are challenging times for brachytherapy physicists, with the rapid clinical implementation of new brachytherapy procedures in a variety of disease sites, and they will be well‐served by this book.
